# Enhanced Production of Bacterial Cellulose from *Miscanthus* as Sustainable Feedstock through Statistical Optimization of Culture Conditions

**DOI:** 10.3390/ijerph19020866

**Published:** 2022-01-13

**Authors:** Jemin Son, Kang Hyun Lee, Taek Lee, Hyun Soo Kim, Weon Ho Shin, Jong-Min Oh, Sang-Mo Koo, Byung Jo Yu, Hah Young Yoo, Chulhwan Park

**Affiliations:** 1 Department of Chemical Engineering, Kwangwoon University, Seoul 01897, Korea; wkqh14@gmail.com (J.S.); tlee@kw.ac.kr (T.L.); 2 Department of Biotechnology, Sangmyung University, Seoul 03016, Korea; oys7158@naver.com; 3 Department of Electronic Engineering, Kwangwoon University, Seoul 01897, Korea; hyunsookim@kw.ac.kr; 4 Department of Electronic Materials Engineering, Kwangwoon University, Seoul 01897, Korea; weonho@kw.ac.kr (W.H.S.); jmOH@kw.ac.kr (J.-M.O.); smkoo@kw.ac.kr (S.-M.K.); 5 Research Institute of Clean Manufacturing System, Korea Institute of Industrial Technology, Cheonan 31056, Korea

**Keywords:** biorefinery, optimization, bacterial cellulose, feedstock, *Miscanthus*, hydrolysate

## Abstract

Biorefineries are attracting attention as an alternative to the petroleum industry to reduce carbon emissions and achieve sustainable development. In particular, because forests play an important role in potentially reducing greenhouse gas emissions to net zero, alternatives to cellulose produced by plants are required. Bacterial cellulose (BC) can prevent deforestation and has a high potential for use as a biomaterial in various industries such as food, cosmetics, and pharmaceuticals. This study aimed to improve BC production from lignocellulose, a sustainable feedstock, and to optimize the culture conditions for *Gluconacetobacter xylinus* using *Miscanthus* hydrolysates as a medium. The productivity of BC was improved using statistical optimization of the major culture parameters which were as follows: temperature, 29 °C; initial pH, 5.1; and sodium alginate concentration, 0.09% (*w*/*v*). The predicted and actual values of BC production in the optimal conditions were 14.07 g/L and 14.88 g/L, respectively, confirming that our prediction model was statistically significant. Additionally, BC production using *Miscanthus* hydrolysates was 1.12-fold higher than in the control group (commercial glucose). Our result indicate that lignocellulose can be used in the BC production processes in the near future.

## 1. Introduction

The reckless use of fossil fuels has accelerated greenhouse gas emissions, leading to global climate change [1,2]. Uncontrolled climate change has resulted in catastrophes, such as environmental pollution, reduced food production, and ecological destruction, and these are factors that threaten societal sustainability and public health [1,2,3]. The United Nations (UN) has established the United Nations Framework Convention on Climate Change (UNFCCC) and adopted treaties such as the Kyoto Protocol and the Paris Agreement to combat climate change through development goals that require carbon emission reduction [1,2,3,4]. As a solution, the concept of replacing fossil fuels with biomass which is a sustainable resource has attracted attention [1,5,6]. Various studies are being conducted globally to design carbon-neutral platforms that produce value-added materials such as biopolymers, biofuels, and biochemicals using the biorefinery concept [1,2,7,8].

Bacterial cellulose (BC) is a natural polymer synthesized by bacteria such as *Gluconacetobacter*, *Pseudomonas*, *Rhizobium*, and *Sarcina* [8,9]. BC can potentially serve as an alternative to plant cellulose (PC) because of its unique properties, such as high water retention capacity, mechanical strength, porosity, elasticity, and biocompatibility [8,10,11]. Unlike PC that contains hemicellulose, lignin, pectin, and ash, BC has a high purity and does not require a separate process to remove impurities [8,10,12]. In addition, the utilization of BC can help prevent environmental pollution by reducing the cutting of trees, which are the major source of PC [8,10,13]. The global BC market was estimated at 250 million US dollar in 2019 and is forecasted to grow to 680 million US dollar by the end of 2025 [14]. Various companies such as CelluForce (Quebec, QC, Canada), FiberLean^®^ Technologies (Orono, ME, USA), and Borregaard ChemCell (Sarpsborg, Norway) produce BC which is used in paper, food, pharmaceutical, and cosmetic industries [15]. However, the high production costs involved in the commercial mass production of BC impose limitations on its usage. The Hestrin–Schramm (HS) medium, which is used mainly for BC production requires large amounts of commercial glucose and glycerol resulting in high production costs [8,10,15,16]. Therefore, for the economical and sustainable production of BC, it is necessary to replace commercial carbon sources, such as glucose and glycerol, with inexpensive and renewable raw materials.

Various studies have investigated the utilization of biomass as a carbon source [8,10,17]. The use of conventional biomass from sources such as corn, wheat, and sugar cane for use as a carbon source entails food-related ethical issues [8,18]. In contrast, the use of *Miscanthus* does not impact food security and has several advantages such as high biomass yields per unit of arable land, the ability to grow easily without requiring pesticides or fertilizers, and a long lifespan [8,19]. The global *Miscanthus* yield is estimated to be approximately 6.6 Mt/year, making it a potential biomass source that is readily available in large quantities [8,20]. In addition, the utilization of *Miscanthus* is expected to reduce anthropogenic CO_2_ emissions and soil erosion but increase the soil carbon content and biodiversity [8,21]. For these reasons, *Miscanthus* is considered to be a reasonable source of biomass for biorefineries.

In our previous study [8], lignocellulosic hydrolysates were used for BC production, and the effect of inhibitors on BC production was investigated. The purpose of this study was to maximize BC production by optimizing the culture conditions using *Miscanthus* hydrolysates as a carbon source. To improve BC production, the culture conditions for *Gluconacetobacter xylinus* ATCC 53524 with regard to the correlation between temperature, initial pH, and sodium alginate (NaAlg) concentration were optimized using a statistical method. 

## 2. Materials and Methods

### 2.1. Materials

Yeast extract, peptone, and agar were purchased from Difco (Detroit, MI, USA). Ammonium sulfate, potassium phosphate monobasic, and magnesium sulfate heptahydrate were purchased from Daejung Chemicals and Metals (Siheung, Korea). Corn steep liquor (CSL) and glucose were purchased from Sigma-Aldrich (St. Louis, MO, USA).

### 2.2. BC Production

*Gluconacetobacter xylinus* ATCC 53524 was pre-cultured in YPD medium (10 g/L yeast extract, 20 g/L peptone, and 20 g/L dextrose) as a seed culture at 30 °C with shaking at 150 rpm for 24 h. The seed suspension (4 mL) was inoculated into 50 mL of the main medium for BC production and cultured at 30 °C and 150 rpm for 4 days. The main medium comprised 40 g/L glucose in *Miscanthus* hydrolysates, 20 g/L CSL, 4 g/L ammonium sulfate, 2 g/L potassium phosphate monobasic, and 0.4 g/L magnesium sulfate heptahydrate. In the control group, the same components were used, except for the carbon source, which was replaced with commercial glucose.

The produced BC was separated from the main medium and washed with 1 N sodium hydroxide at 30 °C for 24 h to remove impurities such as residual medium, cell fragments, and proteins and nucleic acids derived from cells. The BC was next washed with distilled water at 30 °C for 24 h to remove the sodium hydroxide used for washing impurities and dried at 60 °C for 24 h. BC production (g/L) was calculated as follows:

(1) $$\mathrm{BC}\;\mathrm{production}\;[g/L]=\frac{\mathrm{dried}\;\mathrm{BC}\;\mathrm{weight}\;[g]}{\mathrm{volume}\;\mathrm{of}\;\mathrm{culture}\;\mathrm{medium}\;[L]}$$

### 2.3. Experimental Design

The response surface methodology (RSM) is an experimental design that reduces the number of experiments required and provides reliable data based on statistical and mathematical analyses [22]. Central composite design (CCD) is a frequently used technique in RSM, and it provides a predictive model with high accuracy by clearly explaining the correlation between variables [23]. To investigate the optimal conditions for BC production, CCD of RSM was performed using the software Design–Expert 7 (Stat-Ease Inc, MN, USA). Table 1 shows the three variables including temperature, initial pH, and NaAlg concentration, divided into five different levels (−2, −1, 0, 1, and 2). The range of each variable was as follows: temperature (*X*_1_), 20–40 °C; initial pH (*X*_2_), 3–7; and NaAlg concentration (*X*_3_), 0.00–0.16% (*w*/*v*).

Analysis of variance (ANOVA) was performed based on the experimental results to verify the reliability of the predicted model and explain the correlation between the variables. Each variable and its interaction are described by the following quadratic equation:

(2) $$Y=\beta_{0}+\overset{n}{\underset{i=1}{\sum }}\beta_{i}X_{i}+\overset{n}{\underset{i=1}{\sum }}\beta_{ii}X_{i}^{2}+\overset{n-1}{\underset{i=1}{\sum }}\overset{n}{\underset{j=1}{\sum }}\beta_{ij}X_{i}X_{j}$$

where *Y* is the predicted response, *X_i_* and *X_j_* are the independent variables, *β*_0_ is the offset term, *β_i_* is the first-order model coefficient, *β_ii_* is the quadratic coefficient for the variable *i*, and *β_ij_* is the linear model coefficient for the interaction between the variables *i* and *j* [24].

## 3. Results and Discussion

CCD was performed to investigate the optimal culture conditions for *G. xylinus* ATCC 53524 for enhanced BC production. Table 2 shows 20 experiments designed by dividing three independent variables (*X*_1_: temperature, *X*_2_: initial pH, and *X*_3_: NaAlg concentration) into five different levels (−2, −1, 0, 1, and 2) and the experimental results obtained. The range of response (BC production, g/L) was 0.00–14.63 g/L. The experiments were performed six times (Std nos. 15–20) at the center point to confirm reproducibility.

Studies have reported that the addition of NaAlg to the culture medium affects the BC yield, crystallinity index, contact angles, and hydrophilicity [25]. Zhou et al. [26] achieved increased BC production (6.0 g/L) using *Acetobacter xylinum* NUST4.1 by adding 0.04% (*w*/*v*) NaAlg (BC production of the control group without NaAlg was 3.7 g/L). However, according to Cheng et al. [27], the addition of NaAlg at concentrations above 0.2% (*w*/*v*) negatively affected BC production. Therefore, the center point of *X*_3_ was set to 0.08% (*w*/*v*) to accurately evaluate the effect of the NaAlg concentration on BC production.

The model equation for predicting the response was determined using multiple regression analysis of the experimental results as follows:

(3) $$Y=13.92-0.94X_{1}+0.61X_{2}+0.33X_{3}-0.26X_{1}X_{2}-0.34X_{1}X_{3}-0.42X_{2}X_{3}-3.52X_{1}^{2}-2.96X_{2}^{2}-0.51X_{3}^{2}$$

where *Y* is BC production (g/L), and *X*_1_, *X*_2_, and *X*_3_ are the independent variables representing temperature, initial pH, and NaAlg concentration, respectively. 

Table 3 shows the ANOVA results for the quadratic model of BC production. The *F*-value, which indicates the accuracy of the model [28], for the predictive model was 25.28. A value of *p* < 0.05 was considered statistically significant [29], and the results of our predictive model were demonstrated to be statistically significant (*p* < 0.0001). The model terms affecting BC production were found to be *X*_1_, *X*_1_^2^, and *X*_2_^2^; temperature (*X_1_*) was the most significant variable among the three independent variables examined. The statistical acceptability of the predictive model was assessed using the coefficient of determination (R^2^), and a value close to 1 indicates that the experimental response agrees with the predicted response within the designed experimental range [30]. The R^2^ values higher than 0.8 and a difference between R^2^ and adjusted R^2^ not exceeding 0.2 indicate the reliability of the model [31]. The R^2^ and adjusted R^2^ of our model were 0.9579 and 0.9200, respectively, indicating the statistical acceptability of our model.

**Figure 1:**
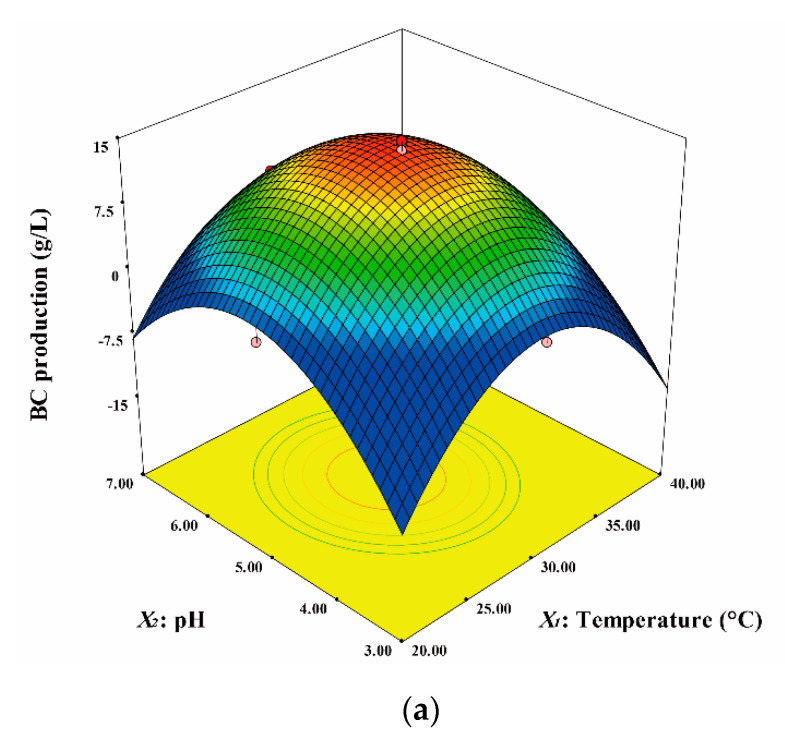

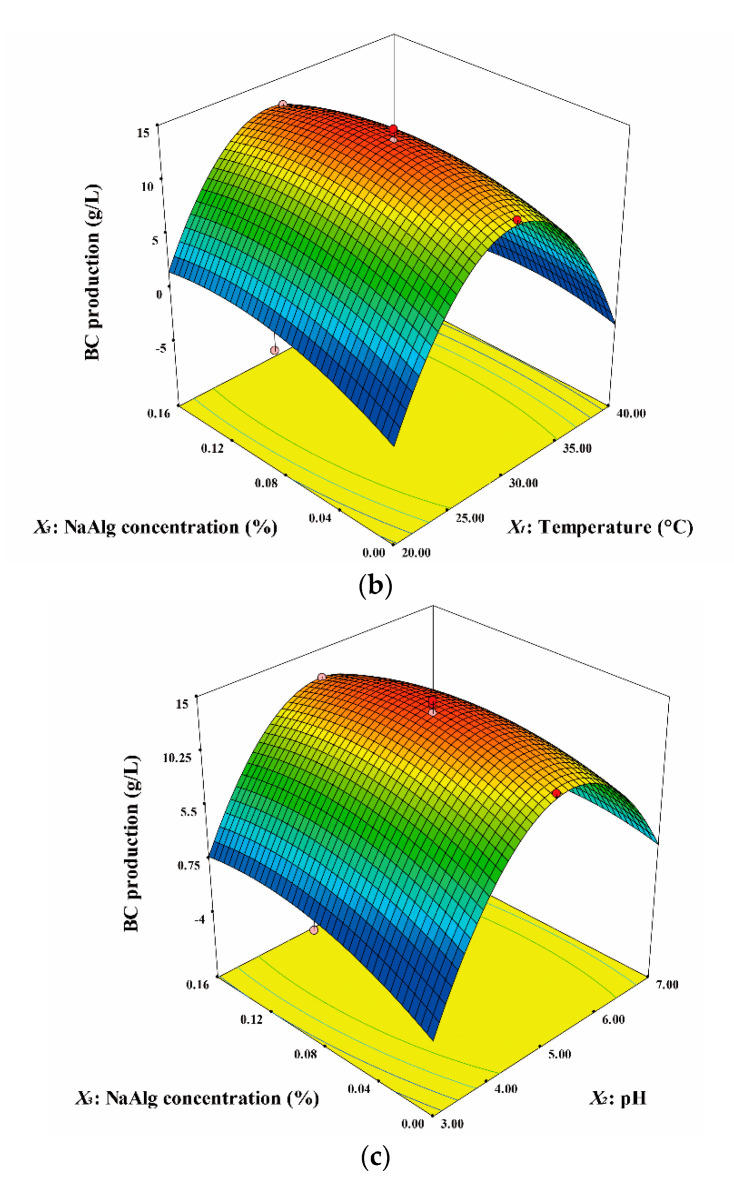
Response surface plots representing the effects of each variable on bacterial cellulose production. (**a**) Effects of temperature and initial pH; (**b**) effects of temperature and NaAlg concentration; (**c**) effects of initial pH and NaAlg concentration.

Figure 1 shows the three-dimensional plots based on Equation (3). Figure 1a shows the effects of temperature and initial pH on BC production when the NaAlg concentration is the center point (*X*_3_ = 0). BC production was maximal when both the temperature and initial pH had values approximately equal to the corresponding values at the center points (temperature of 30 °C and initial pH of 5) and showed a marked decrease when the values of both variables deviated from those at the center point. Figure 1b shows the effects of temperature and NaAlg concentration on BC production when the initial pH is the center point (*X*_2_ = 0). BC production was maximal when the temperature was approximately equal to that at the center point (temperature of 30 °C and NaAlg concentration of 0.08% [*w*/*v*]) and decreased sharply as the temperature varied from that at the center point. The effects of initial pH and NaAlg concentration on BC production are shown in Figure 1c. The results indicate that when the temperature is the center point (*X*_1_ = 0), BC production was maximal when both the variables had values equal to those at the center point (initial pH of 5 and NaAlg concentration of 0.08% [*w*/*v*]). Figure 1b,c show that the NaAlg concentration had no significant effect on BC production compared to the other variables.

Numerical optimization was performed using multiple regression model analysis to derive the optimal culture conditions that can maximize BC production. Table 4 shows the culture conditions derived from the numerical optimization for the predicted and actual BC production values. The optimal culture conditions for enhanced BC production derived by the predictive model were as follows: temperature, 29.24 °C; initial pH, 5.09; and NaAlg concentration, 0.09% (*w*/*v*). The predicted BC production under optimal conditions was 14.07 g/L. To verify the reproducibility of the predictive model, *G. xylinus* ATCC 53524 was cultured under the derived optimal conditions. The relevance of the model was verified by obtaining BC production approximately equal to 14.88 g/L, indicating that the value for the experimentally obtained yield was approximately 94.2%, consistent with that obtained using the prediction model.

In our previous study [8], we determined the phenolic compound contents in *Miscanthus* hydrolysates to be as follows: 0.13 g/L acetic acid, 0.16 g/L formic acid, 0.02 g/L furfural, and 0.05 g/L 5-(hydroxymethyl)furfural. These phenolic compounds have been reported to inhibit microbial growth [32]. However, *Miscanthus* hydrolysates did not inhibit BC production via *G. xylinus* ATCC 53524 fermentation, indicating their potential as a renewable raw material [8]. Therefore, we investigated the optimal culture conditions for improving BC production using *Miscanthus* hydrolysates. BC production obtained from the control group using commercial glucose was 13.26 g/L, whereas that from the group using *Miscanthus* hydrolysates under optimal culture conditions was 14.88 g/L. Thus, the *Miscanthus* hydrolysate group achieved a production equivalent to 112% relative to the production by the control group. A method for calculating the theoretical maximum BC production was shown by Soeiro et al. [33]. With the glucose concentration of the medium used in this study, the theoretical maximum BC production was determined to be about 36 g/L, and BC conversion using *Miscanthus* hydrolysates was about 41% of the theoretical maximum for BC production. BC conversion using *Miscanthus* hydrolysates was greater than that observed in other studies that used hydrolysates of potato peel (32.1%) [34], orange peel (22.0%) [35], and sweet sorghum root (34.9%) [36], indicating that *Miscanthus* is a promising feedstock for BC production.

In a previous study [8], BC production using *Miscanthus* hydrolysates was 97.86% relative to that of the control group. As our previous study [8] focused on the potential utilization of *Miscanthus* hydrolysates as an inexpensive substrate for BC production, we fermented *G. xylinus ATCC 53524* under culture conditions (temperature, 30 °C; initial pH, 6.0; 7 days) that are generally known to promote sufficient growth without considering variables that increase BC production.

We aimed to optimize the culture conditions by considering various variables reported to affect BC production, such as temperature, initial pH, and NaAlg concentration, for economical and sustainable BC production by shortening the culture time. We succeeded in shortening the culture time for BC production from 7 days to 4 days due to the effect of these variables which were not considered previously, and the BC production was determined to be 14.88 g/L. These results are presumably due to the effect of the initial pH and NaAlg. Mikkelsen et al. [37] reported that the optimal pH for BC production by *G. xylinus* ATCC 53524 was 5.0. In addition, according to Zhou et al. [26], the addition of NaAlg not only improved BC yield but also enhanced cell growth of *A. xylinum*. Improvement of BC productivity through optimization of culture conditions will contribute to overcoming the low economic feasibility of biorefinery. Improvement of BC productivity through optimization of culture conditions will contribute to overcoming the low economic feasibility of biorefinery. In particular, our research in which *Miscanthus* hydrolysate was applied as a useful feedstock provides a direction for sustainable and eco-friendly BC production.

## 4. Conclusions

In this study, the optimal culture conditions for *G. xylinus* ATCC 53524 were investigated using statistical methods to improve BC production using *Miscanthus* hydrolysates. The derived optimal culture conditions were as follows: temperature, 29 °C; initial pH, 5.1; and NaAlg concentration, 0.09% (*w*/*v*). Under the derived optimal conditions, the predicted and actual BC yields were 14.07 g/L and 14.88 g/L, respectively. These results demonstrate that our predictive model was statistically significant. In addition, BC production using *Miscanthus* hydrolysates was 1.12-fold greater relative to the control group wherein commercial glucose was used. Taken together, the results of our study for optimizing the culture conditions using *Miscanthus* hydrolysates to enhance BC production is expected to provide useful insights into methods for mitigating global warming and improving public health.

## Figures and Tables

**Table 1:** Variables and the coded variable levels of the central composite design under experimental conditions.

Variables	Unit	Symbol	Coded Variable Levels				
			−2	−1	0	1	2
Temperature	°C	*X*_1_	20	25	30	35	40
Initial pH		*X*_2_	3	4	5	6	7
NaAlg concentration	%, *w*/*v*	*X*_3_	0.00	0.04	0.08	0.12	0.16

**Table 2:** The designed experimental plan and the result values for five-level, three-variable response surface analysis.

Std.	Coded Variable Levels			BC Production (g/L)	
	*X*_1_	*X*_2_	*X*_3_	Actual	Predicted
1	−1	−1	−1	6.98	5.91
2	1	−1	−1	4.64	5.23
3	−1	1	−1	8.75	8.48
4	1	1	−1	4.97	6.78
5	−1	−1	1	9.62	8.09
6	1	−1	1	5.49	6.05
7	−1	1	1	9.30	8.99
8	1	1	1	4.57	5.92
9	−2	0	0	0.00	1.73
10	2	0	0	0.00	−2.02
11	0	−2	0	0.00	0.86
12	0	2	0	4.46	3.31
13	0	0	−2	11.59	11.20
14	0	0	2	12.43	12.53
15	0	0	0	14.36	13.92
16	0	0	0	13.44	13.92
17	0	0	0	14.63	13.92
18	0	0	0	13.16	13.92
19	0	0	0	13.59	13.92
20	0	0	0	14.63	13.92

**Table 3:** Analysis of variance (ANOVA) for the response surface model.

Source	Sum of Squares	DF	Mean Square	*F*-Value	*p*-Value
Model	474.26	9	52.70	25.28	<0.0001
$X_{1}$	14.03	1	14.03	6.73	0.0267
$X_{2}$	5.97	1	5.97	2.86	0.1214
$X_{3}$	1.77	1	1.77	0.85	0.3791
$X_{1}X_{2}$	0.53	1	0.53	0.25	0.6257
$X_{1}X_{3}$	0.94	1	0.94	0.45	0.5182
$X_{2}X_{3}$	1.39	1	1.39	0.67	0.4331
$X_{1}^{2}$	310.76	1	310.76	149.08	<0.0001
$X_{2}^{2}$	220.01	1	220.01	105.55	<0.0001
$X_{3}^{2}$	6.62	1	6.62	3.18	0.1051
Residual	20.85	10	2.08		
Lack of Fit	18.73	5	3.75	8.85	0.0159
Pure Error	2.12	5	0.42		
Cor Total	495.10	19			

Coefficient of determination (R^2^): 0.9579. Adjusted R^2^: 0.9200.

**Table 4:** Optimization of bacterial cellulose production using the regression model.

Parameters	Goal	Importance	Predicted	Actual
Temperature (°C)	In range	–	29.24	29
Initial pH	In range	–	5.09	5.1
NaAlg concentration (%, *w*/*v*)	In range	–	0.09	0.09
Bacterial cellulose production (g/L)	Maximize	3	14.07	14.88

## Data Availability

The data presented in this study are available on request from the corresponding author.
